# Validation of a Model Predicting Anti-infective Lung Penetration in the Epithelial Lining Fluid of Humans

**DOI:** 10.1007/s11095-017-2336-7

**Published:** 2018-01-08

**Authors:** Linda B. S. Aulin, Pyry A. Valitalo, Matthew L. Rizk, Sandra A. G. Visser, Gauri Rao, Piet H. van der Graaf, J. G. Coen van Hasselt

**Affiliations:** 1 Leiden Academic Centre for Drug Research, Leiden University, Einsteinweg 55, 2333 CC Leiden, Netherlands; 2 Orion Corporation Orion Pharma, Kuopio, Finland; 3 Merck & Co. Inc., Kenilworth, New Jersey USA; 4 GSK, King of Prussia, Pennsylvania USA; 5 Eshelman School of Pharmacy, University of North Carolina at Chapel Hill, Chapel Hill, North Carolina USA; 6 Certara, Canterbury, UK

**Keywords:** antibiotics, epithelial lining fluid, lung, pharmacokinetics, prediction

## Introduction

Community and hospital acquired bacterial pneumonias are associated with significant mortality and morbidity (1). It is essential to achieve sufficiently high antibiotic exposure in the epithelial lining fluid (ELF) in order to obtain sufficient efficacy and to prevent selection for resistant or persistent bacterial subpopulations (2). However, antibiotic concentrations in the ELF may be significantly different from concentration in the plasma (3). Hence, the characterization of antibiotic ELF concentrations is important for antibiotics aimed at treating lung infections. Bronchoalveolar lavage (BAL) is most commonly used to quantify antibiotic drug concentrations in humans. However, the technique has significant limitations including the limitation of a single sample per patient given the invasiveness of obtaining the BAL sample (4), and the significant variability between measurements. Approaches to predict lung penetration of antibiotics are thus highly relevant to support informative BAL study design and to support the selection or prioritization of antibiotic candidates.

We previously developed a quantitative structure-pharmacokinetic parameter relationship (QSPKR) model to predict antibiotic lung penetration of several classes of anti-infective agents (5). This model utilized a regularized elastic net regression approach to relate multiple specific chemical structural properties or descriptors to the ratio of the concentration in the ELF to the unbound plasma concentration. The model was trained based on log-transformed clinical ELF and plasma concentration data from 56 unique anti-infective compounds that were extracted from the previous publications of clinical lung penetration studies. The model was validated using a leave-one-out cross validation and by prediction of a limited set of five anti-infective compounds not used for model development. Since then several new clinical lung penetration studies have been published. The aim of this report was to perform a more extensive external validation of the published model to further evaluate its predictive value.

## Methods

We searched the PubMed database and relevant microbiology conference abstracts for clinical studies reporting anti-infective ELF and plasma concentrations in humans between year 2011 and 2017. We also included the five antibiotics used for the original external validation. Antibiotics already present in the training dataset used for the model development were excluded. For each drug identified and included we extracted the non-extrapolated mean AUC_ELF_ and AUC_plasma_ values. The AUC_plasma_ values were converted to unbound concentrations (*f*AUC_plasma_) using the reported protein binding values obtained from the DrugBank database. The AUC_ELF_-*f*AUC_plasma_ ratio was collected and subsequently log-transformed to obtain the clinically observed log ELF/plasma penetration ratio (EPR). Using an R script (included as supplemental material in the original model publication), we generated the same 145 chemical descriptors used for the developed QSPKR model using the R package Rcdk. Subsequently, we applied the original elastic net regression model, without any modifications, to predict the log EPR values for each antibiotic included in the new validation data set, which were compared with the clinically reported log EPRs. This comparison was done by graphically assessing the clinically reported log EPR versus the model predicted log EPR values as well as reporting the percentage of the predictions being outside a 3-fold change from the observations. Additionally we assessed the models capability to characterize drugs ability to penetrate the lungs in a semi-quantitative manner.

## Results

We identified nine anti-infective drugs for which EPRs could be determined and which were not included in the original model. Together with the five antibiotics (arbekacin, GSK2251052, tedizolid, imipenem, peramivir) previously included for the external validation in the original publication the new validation dataset comprised of 14 compounds. The studies for each of these 14 anti-infective agents are summarized in Table I.

**Table I:** Overview of Antibiotics and Associated Clinical Studies Included for the External Validation Analysis. All Studies were Conducted in Healthy Volunteers Except for the Study of Temocillin. Sampling was made with Bronchoalveolar Lavage in ALL Studies Except the Study of Arbekacin and Peramivir for Which Bronchoscopic Microsampling was Utilized

Drug name	Nr of subjects	Nr of time points	Time of last ELF sample (hours)	Study conducted at steady state?	Protein binding (%)	EPR (log EPR)	Reference
Arbekacin	6	8	6	-	6	0.72 (-0.33)	(6)
Avibactam	42	4	8	+	8	0.35 (-1.05)	(7)
Ceftaroline	53	5	12	+	20	0.23 (-1.47)	(8)
Ceftolozane	25	5	8	+	20	0.59 (-0.53)	(9)
Eravacycline	20	4	12	+	83	6.44 (1.86)	(10)
GSK22510052	15	3	12	+	10	0.597 (-0.55)	(11)
Imipenem	16	4	3	+	20	0.53 (-0.63)	(12)
Lefamulin	12	4	8	-	87	5.7 (1.74)	(13)
Omadacycline	42	7	24	+	20	1.84 (0.61)	(14)
Peramivir	6	8	5	-	30 (15)	0.54 (-0.61)	(16)
Relebactam	16	4	3	+	20	0.54 (-0.61)	(12)
Tedizolid	20	4	24	+	89	39.7 (3.68)	(17)
Temocillin	10	10	24	+	75	0.57 (-0.56)	(18)
Vaborbactam	25	5	8	+	33	0.79 (-0.24)	(19)

**Fig. 1:**
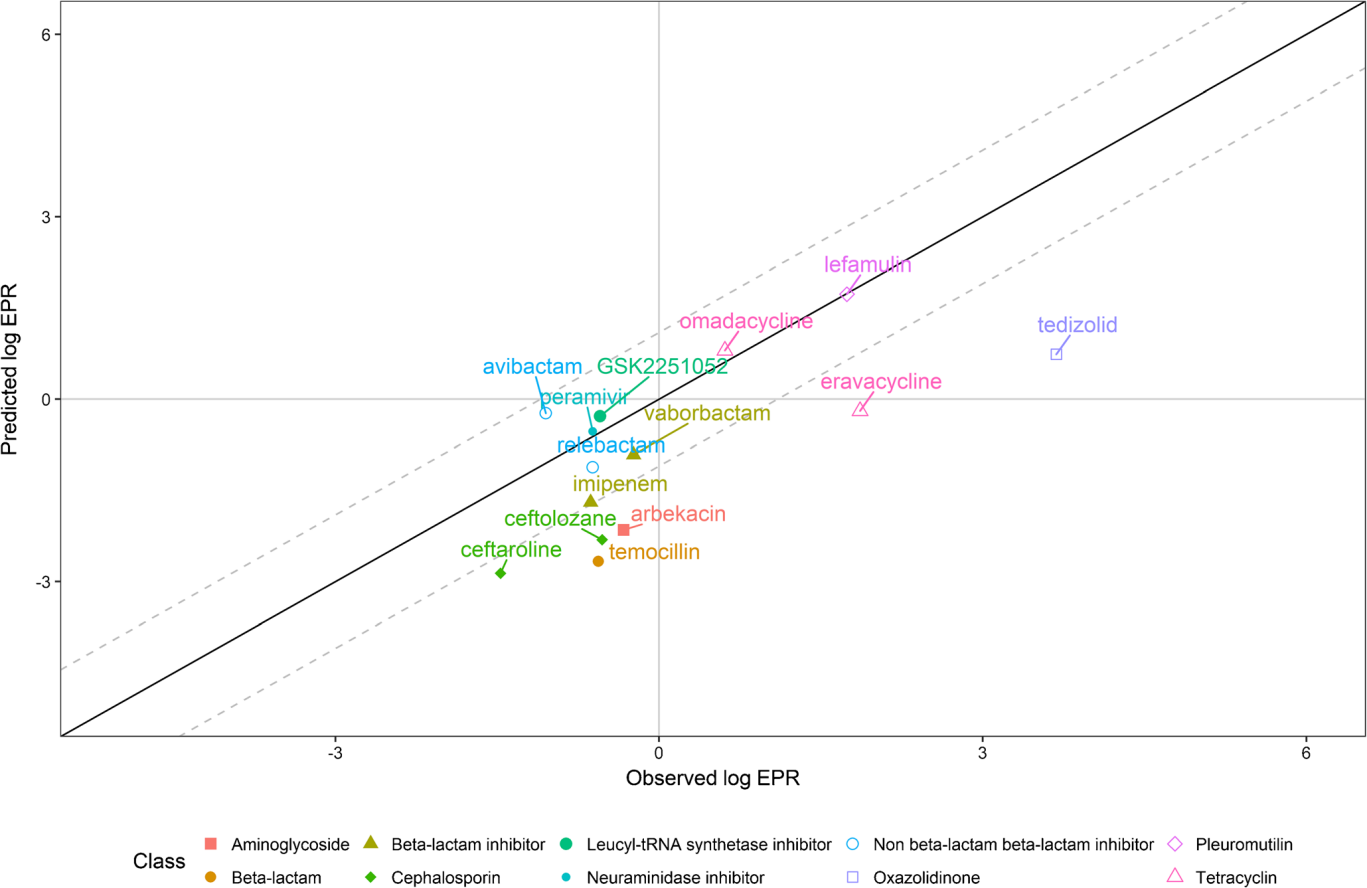
Log-transformed observed versus predicted lung epithelial lining fluid penetration ratios (EPRs) defined as log(AUC_ELF_/*f*AUC_plasma_) stratified by antibiotic class. The dashed line indicates a 3-fold error.

The model predictions for the 14 drugs were in line with the predictive performance reported in the original publication (Fig. 1), with a root mean squared error of 1.42. For 57% (n=8/14) of the drugs the predicted EPRs were within a 3-fold difference from the observations. The model predicted under or over-exposure compared to plasma, i.e. EPR > 1 or EPR < 1 and log EPR > 0 or log EPR < 0 for un-transformed and log-transformed EPR respectively, correctly for 93% (n=13/14) of the drugs. A trend towards under-prediction of the log EPR was observed, particularly for eravacycline, temocillin, and tedizolid.

## Discussion

The external validation analysis of the previous developed QSPKR lung penetration model has shown a predictive performance in line with the reported prediction performance of the original publication. In the current validation the number of antibiotics outside a 3-fold prediction error for un-transformed ERP was 43% whereas in the original publication it was 20% for the small external validation set and 39% for the leave-one-cross validation analysis of the training dataset. Notably, as no model development was performed at this stage, no leave-one-cross validation analysis was conducted and thus no comparable metric was obtained.

Three classes of anti-infective drugs were included in this validation that were not used for the training of the original model, i.e. the leucyl-tRNA synthetase inhibitor GSK2251052, the non-beta-lactam beta-lactam inhibitors avibactam and relebactam, and the pleuromutilin antibiotic lefamulin. These new-in-class anti-infective agents were well predicted, within a 2-fold difference from the observed EPRs.

Eravacycline was associated with substantial lung penetration compared to plasma concentrations, with a clinically observed ERP of 6.44, but was miss-classified by the model as giving under-exposure (EPR<1), indicating that some of the model predictions should be interpreted with caution for compounds that are structurally related to this drug. The model did however correctly classify all drugs associated with under-exposure in the ELF compared to plasma. This suggests the clinical applicability of the model as a tool to inform when dose-adjustments may be warranted and guide in the adjustment for clinically relevant exposure, which could improve treatment and decrease resistance development.

We were not able to identify clear chemical structure characteristics different from the training set that could explain the misclassification of eravacycline or the high predictive errors associated with eravacycline, temocillin, and tedizolid. The three drugs are all relatively highly bound to plasma proteins (≥ 75%), which is not currently included in the model. However, during a post-hoc residual analysis, which included all drugs present in the validation dataset, no correlation was found between protein binding and residuals. Antibiotic class, molecular descriptors, and factors relating to study design were considered in addition to protein binding. However, no strong correlations were found in this analysis. Worth considering is that the ability of detecting correlations was limited due to the small size of the dataset. In the validation dataset eravacycline and tedizolid are the only drugs containing fluorine, while in the training dataset all fluorine containing drugs were primarily fluoroquinolones. Additionally, temocillin was the only drug studied on patient with lung infections and not healthy volunteers. Infection could affect the permeability of the drug to the lungs, i.e. the EPR, contributing to prediction error. In the original dataset difference in clinical observed EPR could be seen between diseases states for some of the drugs. No other study design aspect, such as size of study cohort or sampling technique, could be linked to the under-predictions.

We expect that increasing the mechanistic aspects of this model could improve the model predictions. Currently, plasma protein binding is not explicitly included as a predictor in the model but potentially its consideration could improve the predictions. However, the maximal possible quality of this predictive model is partially limited by the high variability of the ELF concentrations obtained by BAL sampling. The method is associated with inherent uncertainty that is partially related to indirect quantification, possible contamination from cellular release, and technical errors (3), as well as only obtaining a single sample per individual. A more novel and promising sampling technique is bronchoscopic microsampling (BMS), used in 2 of the 14 studies (6,16). The technique is less invasive than BAL and allows for direct repeated measurements of drug concentrations in the ELF over time (20).

## Conclusion

Although the log EPR predictions by the QSPKR model are still associated with a significant error, we nonetheless expect that this QSPKR model is of relevance to determine the expected magnitude of lung penetration on a semi-quantitative basis, i.e. under- or over-exposure, or comparable exposure to plasma. Specifically, the model has relevance to support informative study design of pharmacokinetic studies (21), potentially in conjunction with population pharmacokinetic models (22). Future studies to predict the EPR may benefit from a combined mechanistic PBPK approach to enable prediction of non-steady state ELF pharmacokinetics, while supported by a QSPKR model for estimation of the partitioning coefficients.
